# Supplementary material for: Endophytic and epiphytic metabarcoding reveals fungal communities on cashew phyllosphere in Kenya
Source: PLoS One. 2024 Jul 17;19(7):e0305600. doi: 10.1371/journal.pone.0305600 (PMC11253924; doi:10.1371/journal.pone.0305600)
Supplement: S1 File — (PPTX) [file pone.0305600.s001.pptx]

## Slide 1
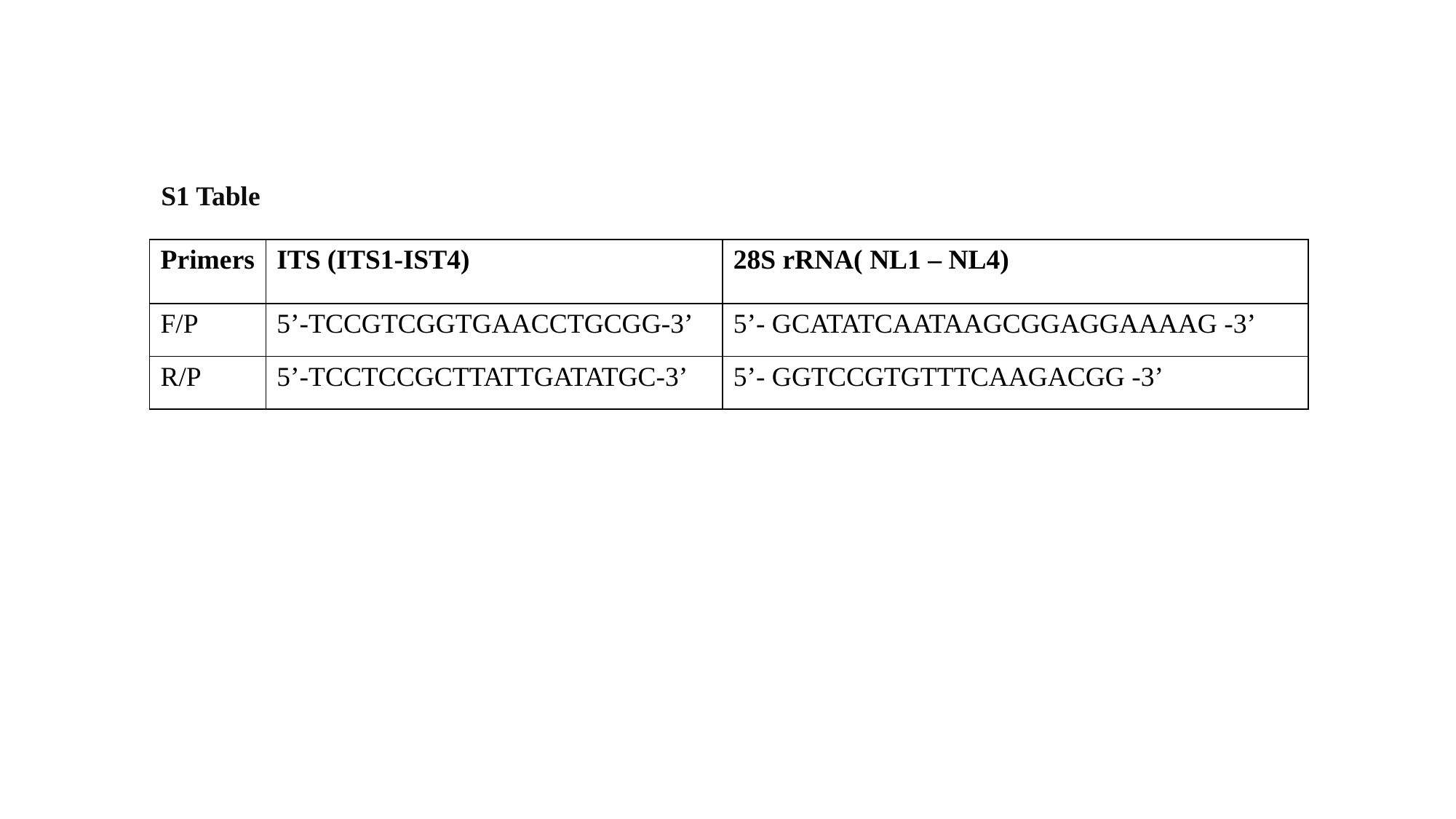

S1 Table
| Primers | ITS (ITS1-IST4) | 28S rRNA( NL1 – NL4) |
| --- | --- | --- |
| F/P | 5’-TCCGTCGGTGAACCTGCGG-3’ | 5’- GCATATCAATAAGCGGAGGAAAAG -3’ |
| R/P | 5’-TCCTCCGCTTATTGATATGC-3’ | 5’- GGTCCGTGTTTCAAGACGG -3’ |

## Slide 2
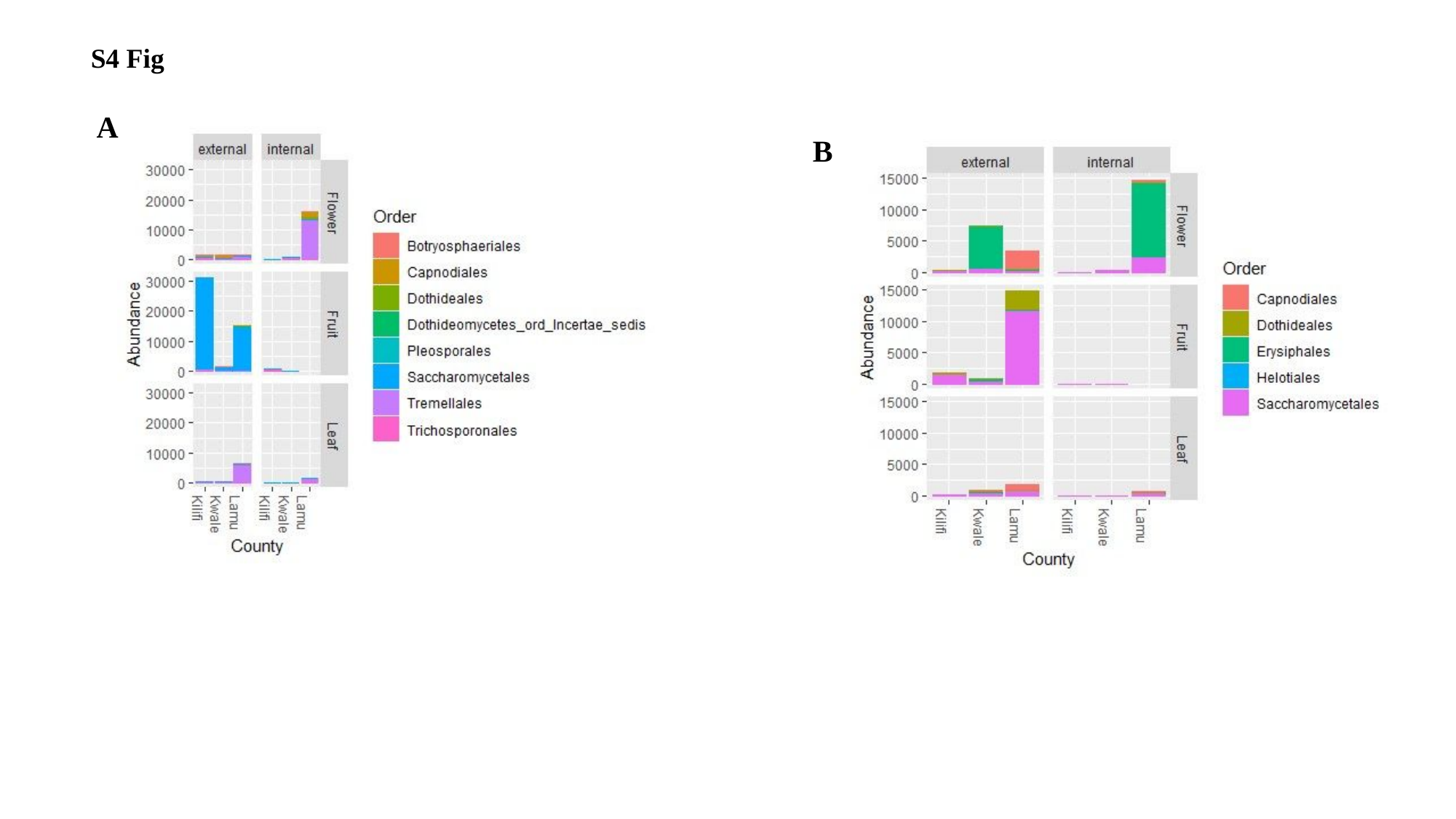

S4 Fig
A
B

## Slide 3
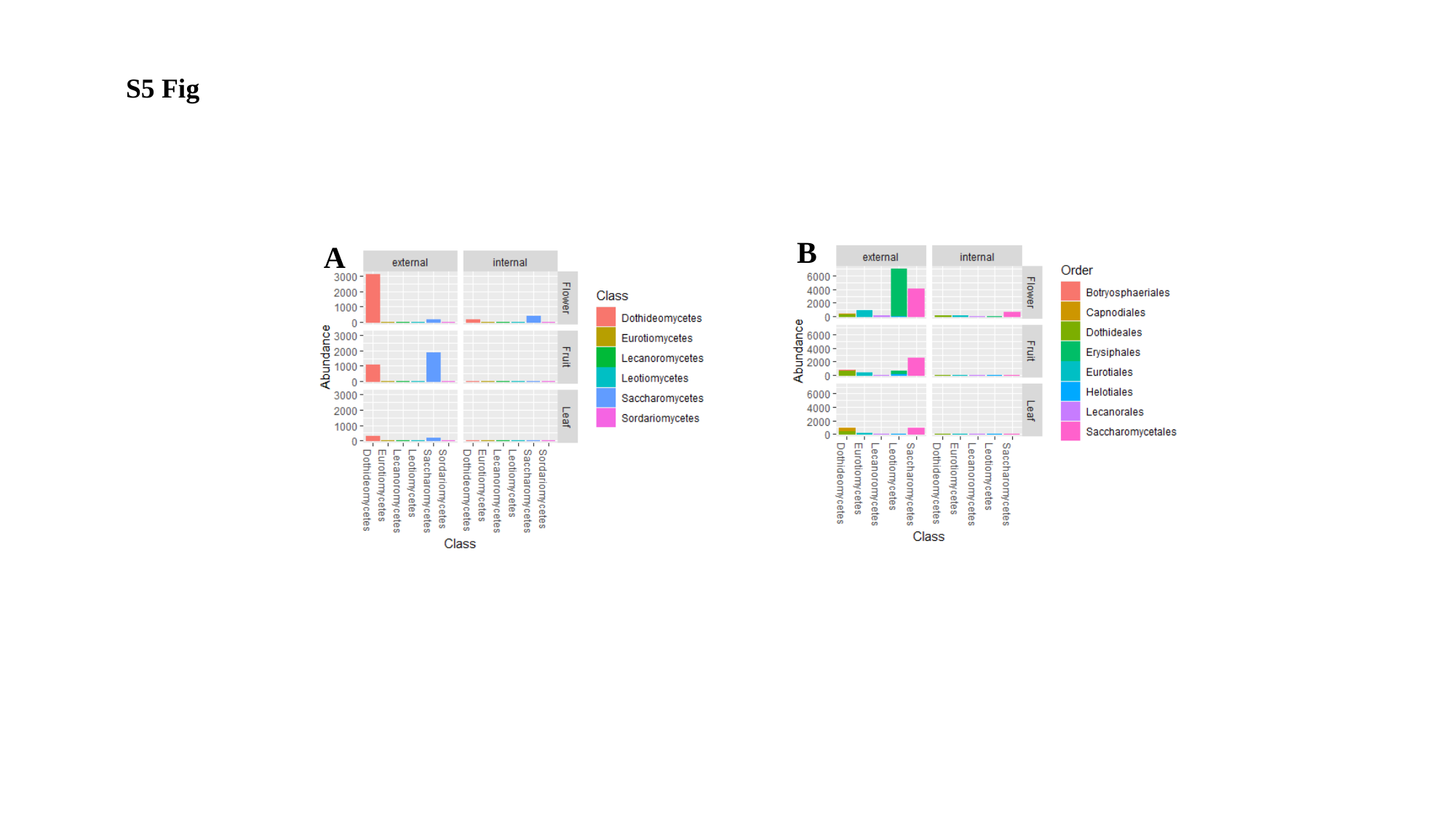

S5 Fig
B
A

## Slide 4
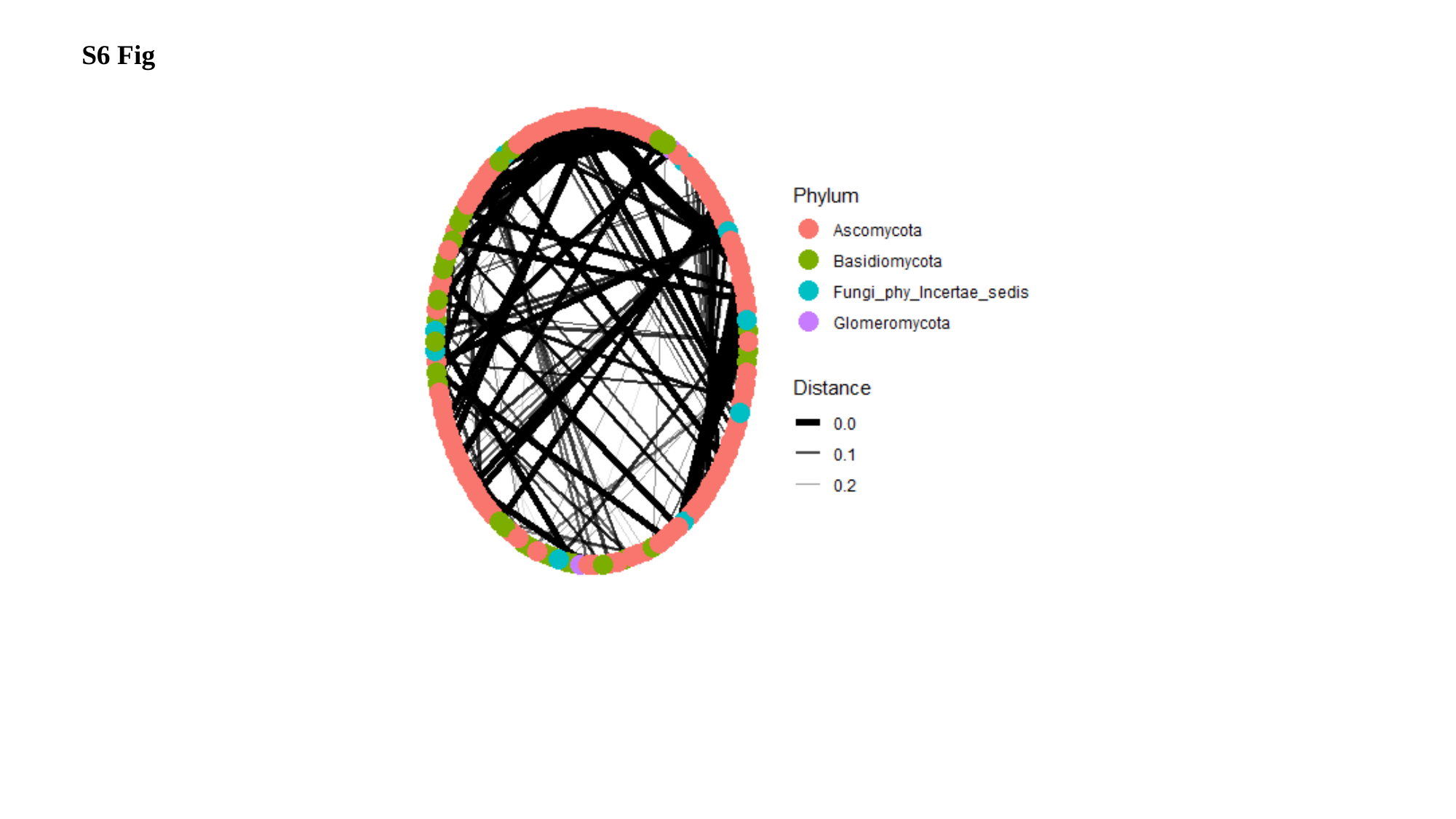

S6 Fig

## Slide 5
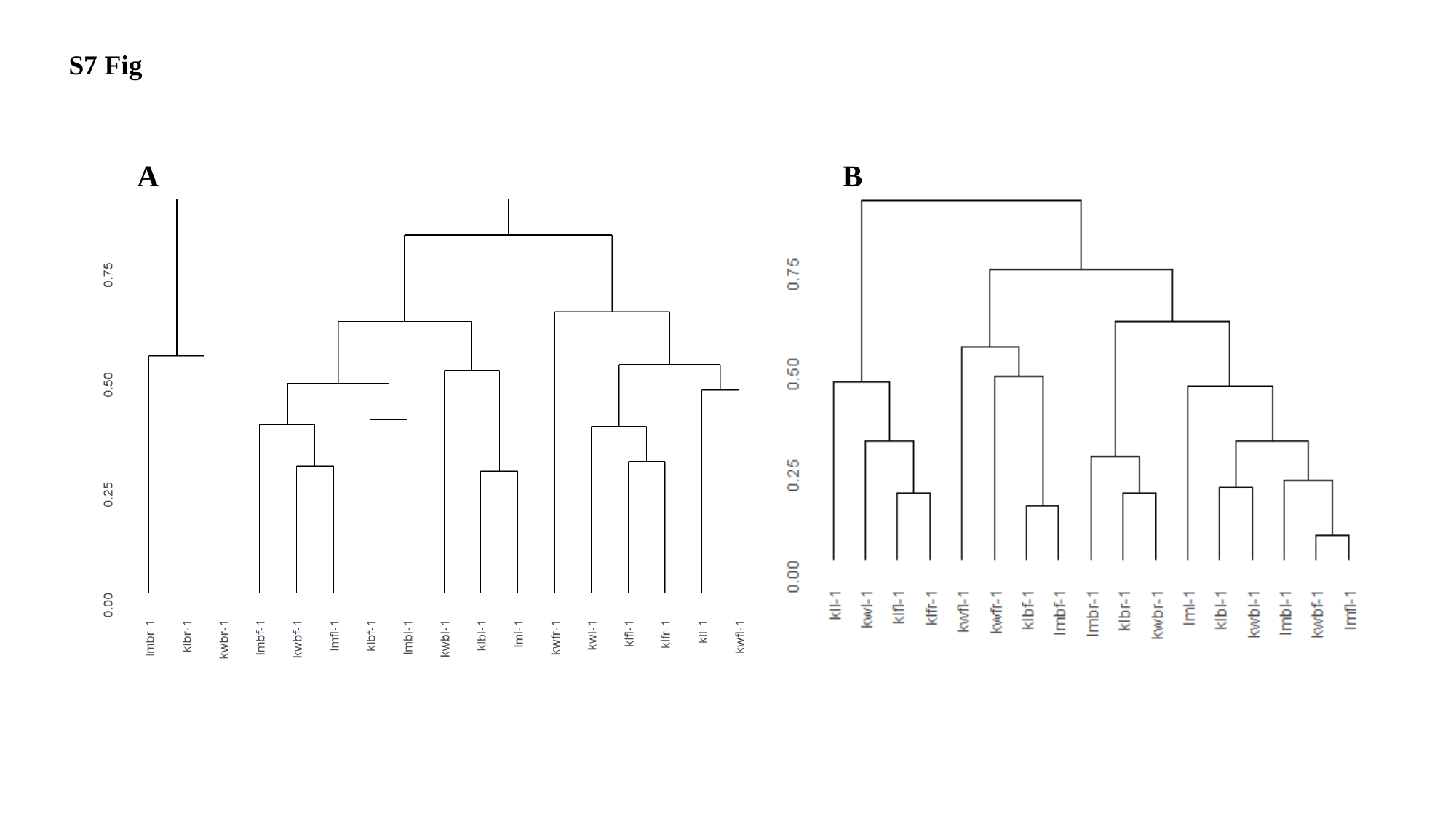

S7 Fig
A
B

## Slide 6
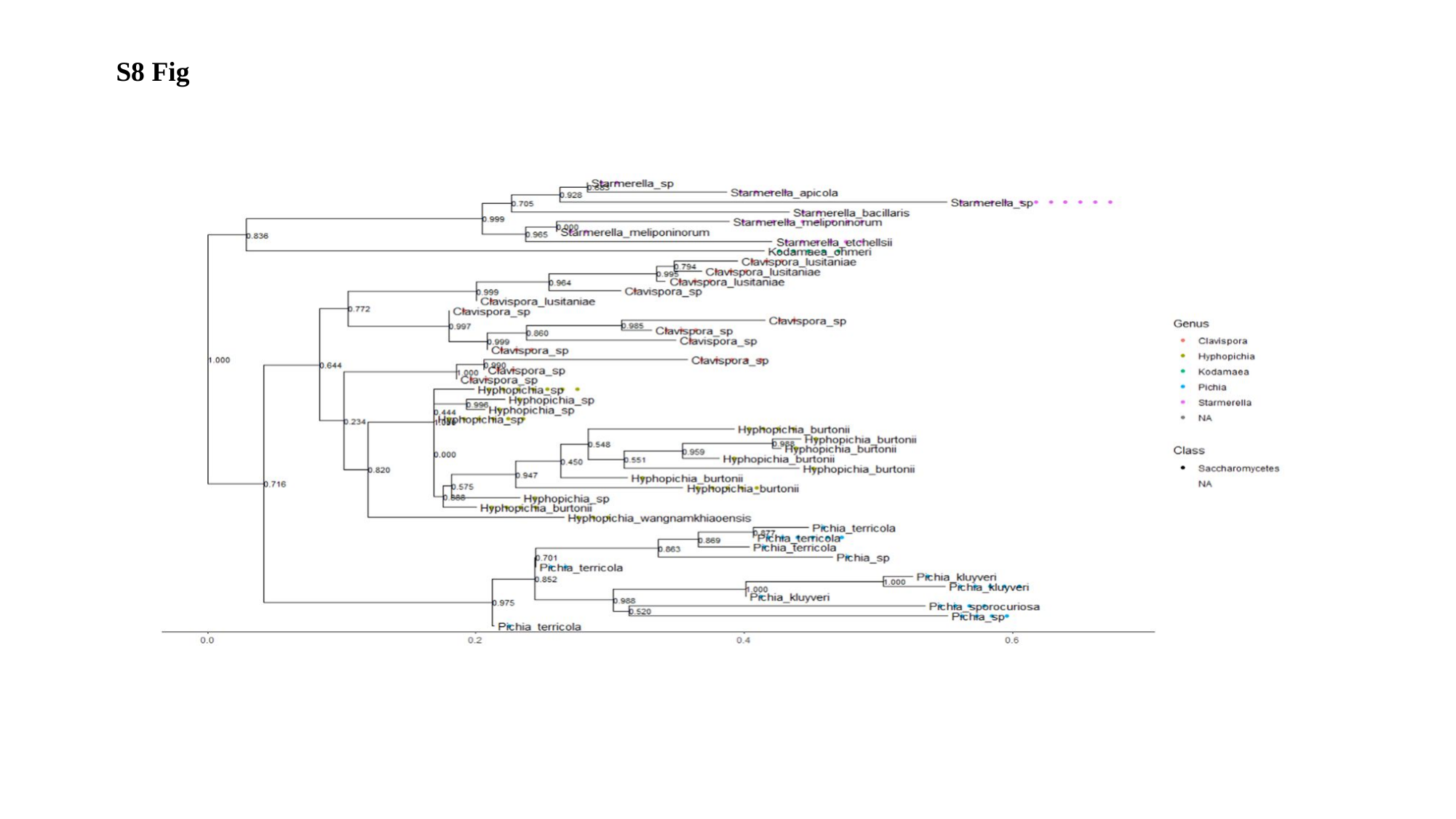

S8 Fig

## Slide 7
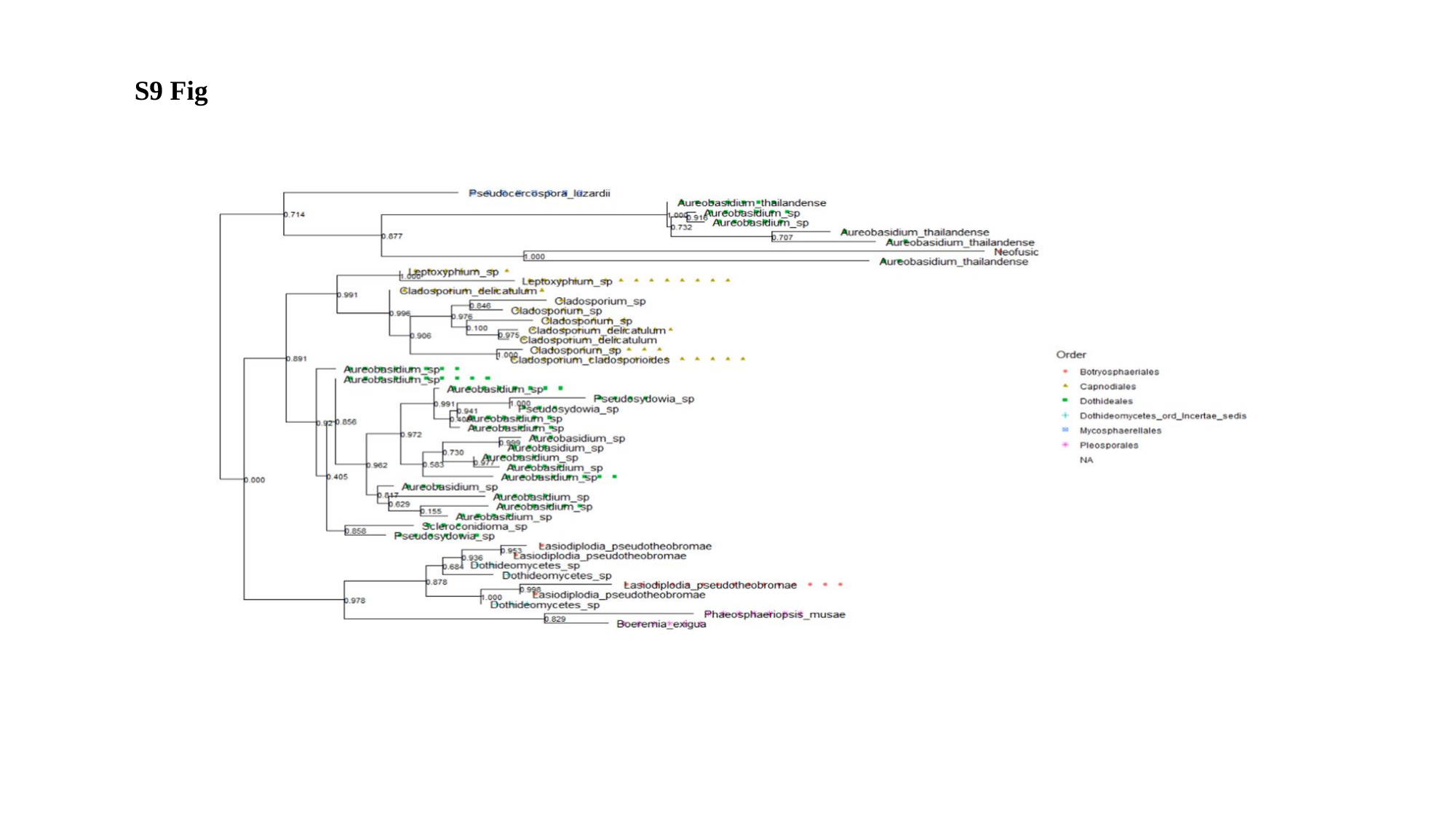

S9 Fig
