# Supplementary material for: Endophytic and epiphytic metabarcoding reveals fungal communities on cashew phyllosphere in Kenya
Source: PLoS One. 2024 Jul 17;19(7):e0305600. doi: 10.1371/journal.pone.0305600 (PMC11253924; doi:10.1371/journal.pone.0305600)
Supplement: S2 File — (DOCX) [file pone.0305600.s002.docx]

**S2 Table**

| **Sample.ID** | **klbf.1** | **klbl.1** | **klbr.1** | **klfl.1** | **klfr.1** | **kll.1** | **kwbf.1** | **kwbl.1** | **kwbr.1** | **kwfl.1** | **kwfr.1** | **kwl.1** | **lmbf.1** | **lmbl.1** | **lmbr.1** | **lmfl.1** | **lml.1** | **Total_OTUs** |
| --- | --- | --- | --- | --- | --- | --- | --- | --- | --- | --- | --- | --- | --- | --- | --- | --- | --- | --- |
| otu1 | 0 | 0 | 280 | 0 | 0 | 0 | 0 | 0 | 349 | 0 | 0 | 0 | 2 | 6986 | 126881 | 0 | 2241 | 273478 |
| otu10 | 174 | 20 | 101 | 0 | 0 | 3 | 270 | 17 | 72 | 0 | 0 | 0 | 536 | 108 | 9 | 7582 | 27 | 17838 |
| otu100 | 0 | 0 | 13 | 0 | 0 | 0 | 0 | 0 | 2 | 0 | 0 | 0 | 0 | 46 | 151 | 0 | 70 | 564 |
| otu101 | 34 | 0 | 0 | 0 | 0 | 0 | 1 | 0 | 0 | 0 | 0 | 4 | 1 | 0 | 0 | 7 | 0 | 94 |
| otu102 | 0 | 0 | 0 | 0 | 0 | 0 | 0 | 0 | 0 | 0 | 0 | 0 | 0 | 0 | 231 | 0 | 0 | 462 |
| otu103 | 0 | 0 | 13 | 0 | 0 | 0 | 0 | 0 | 0 | 0 | 0 | 0 | 0 | 17 | 130 | 0 | 0 | 320 |
| otu104 | 0 | 0 | 0 | 0 | 0 | 0 | 0 | 0 | 0 | 0 | 0 | 0 | 0 | 0 | 132 | 0 | 0 | 264 |
| otu105 | 0 | 0 | 0 | 0 | 0 | 0 | 0 | 0 | 0 | 0 | 0 | 0 | 0 | 0 | 96 | 0 | 0 | 192 |
| otu106 | 0 | 0 | 78 | 0 | 0 | 0 | 24 | 0 | 9 | 0 | 0 | 0 | 20 | 1 | 0 | 249 | 0 | 762 |
| otu107 | 0 | 0 | 0 | 0 | 0 | 0 | 0 | 0 | 3 | 0 | 0 | 0 | 0 | 0 | 52 | 0 | 0 | 110 |
| otu108 | 0 | 0 | 0 | 0 | 0 | 0 | 0 | 0 | 0 | 0 | 0 | 0 | 0 | 0 | 87 | 0 | 0 | 174 |
| otu109 | 9 | 9 | 0 | 0 | 0 | 0 | 0 | 68 | 7 | 0 | 0 | 0 | 0 | 12 | 1 | 14 | 2 | 244 |
| otu11 | 131 | 3 | 1092 | 0 | 0 | 0 | 6 | 2 | 27 | 59 | 0 | 0 | 12 | 612 | 232 | 172 | 54 | 4804 |
| otu110 | 0 | 0 | 0 | 0 | 0 | 0 | 0 | 0 | 10 | 0 | 0 | 0 | 0 | 0 | 114 | 0 | 0 | 248 |
| otu111 | 0 | 0 | 112 | 0 | 0 | 0 | 0 | 0 | 0 | 0 | 0 | 0 | 0 | 0 | 0 | 0 | 0 | 224 |
| otu112 | 0 | 0 | 1 | 0 | 0 | 0 | 95 | 0 | 11 | 0 | 0 | 0 | 6 | 0 | 0 | 81 | 0 | 388 |
| otu113 | 11 | 0 | 0 | 0 | 0 | 0 | 109 | 0 | 39 | 0 | 1 | 0 | 1 | 4 | 3 | 17 | 0 | 370 |
| otu114 | 32 | 1 | 0 | 0 | 0 | 0 | 0 | 0 | 0 | 0 | 0 | 0 | 0 | 0 | 0 | 0 | 0 | 66 |
| otu115 | 0 | 0 | 0 | 0 | 0 | 0 | 0 | 0 | 0 | 0 | 0 | 0 | 0 | 0 | 100 | 0 | 0 | 200 |
| otu116 | 0 | 0 | 0 | 5 | 33 | 0 | 0 | 2 | 0 | 2 | 0 | 0 | 0 | 0 | 0 | 0 | 0 | 84 |
| otu117 | 0 | 0 | 0 | 0 | 0 | 0 | 0 | 0 | 0 | 0 | 0 | 0 | 0 | 0 | 945 | 0 | 0 | 1890 |
| otu118 | 0 | 0 | 0 | 0 | 0 | 0 | 0 | 0 | 0 | 0 | 0 | 0 | 0 | 0 | 13 | 51 | 0 | 128 |
| otu119 | 0 | 0 | 0 | 0 | 0 | 0 | 0 | 0 | 0 | 0 | 0 | 0 | 0 | 0 | 234 | 0 | 0 | 468 |
| otu12 | 222 | 140 | 196 | 0 | 0 | 4 | 45 | 288 | 18 | 98 | 0 | 7 | 394 | 5754 | 7 | 3111 | 1322 | 23212 |
| otu120 | 0 | 0 | 0 | 0 | 0 | 0 | 0 | 0 | 0 | 0 | 0 | 0 | 0 | 0 | 32 | 0 | 0 | 64 |
| otu121 | 0 | 0 | 56 | 0 | 0 | 0 | 0 | 0 | 0 | 0 | 0 | 0 | 0 | 0 | 1 | 0 | 0 | 114 |
| otu122 | 0 | 4 | 0 | 0 | 0 | 0 | 2 | 0 | 1 | 0 | 0 | 0 | 0 | 0 | 0 | 48 | 2 | 114 |
| otu123 | 0 | 0 | 0 | 0 | 0 | 0 | 0 | 0 | 0 | 0 | 0 | 0 | 0 | 0 | 83 | 0 | 0 | 166 |
| otu124 | 0 | 0 | 0 | 0 | 0 | 0 | 0 | 0 | 0 | 0 | 0 | 0 | 0 | 0 | 52 | 0 | 0 | 104 |
| otu125 | 0 | 0 | 4 | 0 | 0 | 0 | 0 | 0 | 0 | 0 | 0 | 0 | 0 | 0 | 83 | 0 | 0 | 174 |
| otu126 | 0 | 0 | 81 | 0 | 0 | 0 | 0 | 0 | 0 | 0 | 0 | 0 | 0 | 0 | 0 | 0 | 0 | 162 |
| otu127 | 0 | 0 | 0 | 0 | 0 | 0 | 0 | 0 | 0 | 0 | 0 | 0 | 0 | 0 | 36 | 0 | 0 | 72 |
| otu128 | 0 | 0 | 29 | 0 | 0 | 0 | 0 | 0 | 7 | 0 | 0 | 0 | 0 | 116 | 442 | 0 | 26 | 1240 |
| otu129 | 0 | 0 | 0 | 0 | 0 | 0 | 0 | 0 | 6 | 0 | 0 | 0 | 0 | 0 | 42 | 0 | 0 | 96 |
| otu13 | 779 | 75 | 16 | 14 | 50 | 0 | 873 | 104 | 358 | 113 | 1 | 8 | 181 | 374 | 26 | 1391 | 16 | 8758 |
| otu130 | 0 | 0 | 0 | 0 | 0 | 0 | 0 | 0 | 0 | 0 | 0 | 0 | 0 | 0 | 92 | 0 | 0 | 184 |
| otu131 | 0 | 0 | 0 | 0 | 0 | 0 | 0 | 0 | 0 | 0 | 0 | 0 | 0 | 0 | 0 | 30 | 0 | 60 |
| otu132 | 0 | 0 | 75 | 0 | 0 | 0 | 0 | 0 | 0 | 0 | 0 | 0 | 0 | 0 | 0 | 0 | 0 | 150 |
| otu133 | 0 | 0 | 25 | 0 | 0 | 0 | 0 | 0 | 0 | 0 | 0 | 0 | 0 | 0 | 0 | 0 | 0 | 50 |
| otu134 | 0 | 0 | 45 | 0 | 0 | 0 | 6 | 0 | 0 | 0 | 0 | 0 | 0 | 8 | 1 | 0 | 0 | 120 |
| otu135 | 0 | 0 | 3 | 0 | 0 | 0 | 11 | 0 | 1 | 0 | 0 | 0 | 6 | 0 | 0 | 93 | 0 | 228 |
| otu136 | 0 | 0 | 2 | 0 | 0 | 0 | 0 | 0 | 0 | 0 | 0 | 0 | 0 | 0 | 183 | 0 | 0 | 370 |
| otu137 | 0 | 0 | 2 | 0 | 0 | 0 | 0 | 0 | 1 | 0 | 0 | 0 | 0 | 0 | 69 | 0 | 0 | 144 |
| otu138 | 2 | 0 | 1 | 0 | 0 | 0 | 13 | 0 | 0 | 0 | 0 | 0 | 2 | 2 | 0 | 161 | 0 | 362 |
| otu139 | 0 | 0 | 41 | 0 | 0 | 0 | 0 | 0 | 0 | 0 | 0 | 0 | 0 | 0 | 0 | 0 | 0 | 82 |
| otu14 | 0 | 0 | 0 | 0 | 0 | 0 | 0 | 0 | 1 | 0 | 0 | 0 | 0 | 0 | 3284 | 0 | 0 | 6570 |
| otu140 | 0 | 0 | 0 | 0 | 0 | 0 | 0 | 0 | 0 | 0 | 0 | 0 | 0 | 48 | 0 | 49 | 4 | 202 |
| otu141 | 0 | 0 | 20 | 0 | 0 | 0 | 0 | 0 | 0 | 0 | 0 | 0 | 0 | 0 | 0 | 0 | 0 | 40 |
| otu142 | 1 | 0 | 0 | 0 | 0 | 0 | 0 | 20 | 0 | 0 | 0 | 0 | 0 | 0 | 0 | 0 | 1 | 44 |
| otu143 | 0 | 0 | 9 | 0 | 0 | 0 | 0 | 0 | 4 | 0 | 0 | 0 | 0 | 0 | 27 | 4 | 0 | 88 |
| otu144 | 0 | 0 | 10 | 0 | 0 | 0 | 0 | 0 | 3 | 0 | 0 | 0 | 0 | 3 | 228 | 0 | 0 | 488 |
| otu145 | 0 | 0 | 0 | 0 | 0 | 0 | 0 | 0 | 0 | 0 | 0 | 0 | 0 | 0 | 37 | 0 | 0 | 74 |
| otu146 | 0 | 0 | 0 | 0 | 0 | 0 | 0 | 0 | 0 | 0 | 0 | 0 | 0 | 1 | 40 | 0 | 0 | 82 |
| otu147 | 18 | 7 | 0 | 0 | 0 | 0 | 1 | 0 | 13 | 6 | 0 | 0 | 0 | 1 | 0 | 0 | 2 | 96 |
| otu148 | 0 | 0 | 0 | 0 | 0 | 0 | 0 | 0 | 0 | 0 | 0 | 0 | 0 | 0 | 28 | 0 | 0 | 56 |
| otu149 | 0 | 3 | 0 | 0 | 0 | 0 | 0 | 0 | 0 | 0 | 0 | 0 | 0 | 0 | 2 | 0 | 11 | 32 |
| otu15 | 0 | 0 | 344 | 0 | 0 | 0 | 1 | 0 | 72 | 0 | 0 | 0 | 0 | 0 | 1238 | 12 | 0 | 3334 |
| otu150 | 0 | 0 | 0 | 0 | 0 | 0 | 0 | 0 | 0 | 0 | 0 | 0 | 0 | 40 | 0 | 3 | 13 | 112 |
| otu151 | 0 | 0 | 0 | 0 | 0 | 0 | 0 | 0 | 0 | 0 | 0 | 0 | 0 | 0 | 106 | 0 | 0 | 212 |
| otu152 | 1 | 1 | 0 | 0 | 0 | 0 | 17 | 0 | 2 | 0 | 0 | 0 | 15 | 1 | 0 | 3 | 0 | 80 |
| otu153 | 0 | 0 | 0 | 0 | 0 | 0 | 0 | 0 | 0 | 0 | 0 | 0 | 6 | 0 | 12 | 0 | 0 | 36 |
| otu154 | 0 | 2 | 0 | 0 | 0 | 0 | 0 | 0 | 0 | 0 | 0 | 0 | 0 | 30 | 0 | 0 | 6 | 76 |
| otu155 | 0 | 0 | 0 | 0 | 0 | 0 | 0 | 0 | 0 | 0 | 0 | 0 | 0 | 0 | 112 | 0 | 0 | 224 |
| otu156 | 0 | 0 | 0 | 0 | 0 | 0 | 0 | 0 | 1 | 0 | 0 | 0 | 0 | 0 | 41 | 0 | 0 | 84 |
| otu157 | 0 | 0 | 0 | 0 | 0 | 0 | 0 | 0 | 0 | 0 | 0 | 0 | 0 | 0 | 12 | 0 | 0 | 24 |
| otu158 | 0 | 0 | 0 | 0 | 0 | 0 | 63 | 0 | 56 | 0 | 0 | 0 | 3 | 0 | 0 | 12 | 0 | 268 |
| otu159 | 0 | 0 | 0 | 0 | 0 | 1 | 0 | 1 | 0 | 0 | 0 | 0 | 1 | 9 | 0 | 17 | 1 | 60 |
| otu16 | 14 | 12 | 88 | 15 | 0 | 0 | 0 | 0 | 124 | 0 | 0 | 0 | 134 | 0 | 1437 | 351 | 9 | 4368 |
| otu160 | 2 | 0 | 3 | 0 | 0 | 0 | 233 | 1 | 11 | 0 | 0 | 0 | 5 | 0 | 0 | 426 | 0 | 1362 |
| otu161 | 0 | 0 | 0 | 0 | 0 | 0 | 0 | 0 | 0 | 0 | 0 | 0 | 0 | 0 | 27 | 0 | 0 | 54 |
| otu162 | 1 | 0 | 0 | 0 | 0 | 0 | 32 | 0 | 0 | 0 | 0 | 0 | 5 | 0 | 0 | 71 | 0 | 218 |
| otu163 | 0 | 0 | 16 | 0 | 0 | 0 | 0 | 0 | 1 | 0 | 0 | 0 | 0 | 12 | 139 | 0 | 3 | 342 |
| otu164 | 0 | 0 | 0 | 0 | 0 | 0 | 0 | 0 | 0 | 0 | 0 | 0 | 0 | 0 | 87 | 0 | 0 | 174 |
| otu165 | 0 | 0 | 0 | 0 | 0 | 0 | 0 | 0 | 0 | 0 | 0 | 0 | 0 | 0 | 32 | 0 | 0 | 64 |
| otu166 | 0 | 0 | 0 | 0 | 0 | 0 | 0 | 0 | 1 | 0 | 0 | 0 | 1 | 0 | 11 | 14 | 0 | 54 |
| otu167 | 0 | 0 | 10 | 0 | 0 | 0 | 0 | 0 | 0 | 0 | 0 | 0 | 0 | 29 | 116 | 0 | 60 | 430 |
| otu168 | 0 | 0 | 0 | 0 | 0 | 0 | 0 | 0 | 0 | 0 | 0 | 0 | 0 | 0 | 58 | 0 | 0 | 116 |
| otu169 | 0 | 0 | 0 | 0 | 0 | 0 | 0 | 0 | 0 | 0 | 0 | 0 | 7 | 0 | 10 | 4 | 3 | 48 |
| otu17 | 0 | 0 | 9 | 0 | 0 | 0 | 0 | 0 | 15 | 0 | 0 | 0 | 0 | 0 | 7279 | 0 | 0 | 14606 |
| otu170 | 0 | 0 | 0 | 0 | 0 | 0 | 0 | 0 | 0 | 0 | 0 | 0 | 0 | 0 | 0 | 20 | 0 | 40 |
| otu171 | 0 | 0 | 0 | 0 | 0 | 0 | 0 | 0 | 0 | 0 | 0 | 0 | 0 | 0 | 36 | 0 | 0 | 72 |
| otu172 | 17 | 0 | 0 | 0 | 0 | 0 | 0 | 0 | 0 | 0 | 0 | 0 | 0 | 0 | 0 | 0 | 0 | 34 |
| otu173 | 0 | 0 | 0 | 0 | 0 | 0 | 34 | 0 | 53 | 0 | 0 | 0 | 0 | 0 | 0 | 6 | 0 | 186 |
| otu174 | 0 | 0 | 17 | 0 | 0 | 0 | 0 | 0 | 0 | 0 | 0 | 0 | 0 | 4 | 27 | 0 | 0 | 96 |
| otu175 | 0 | 0 | 0 | 0 | 0 | 0 | 0 | 0 | 0 | 0 | 0 | 0 | 15 | 0 | 0 | 0 | 0 | 30 |
| otu176 | 0 | 0 | 0 | 0 | 0 | 0 | 0 | 0 | 0 | 14 | 0 | 0 | 0 | 0 | 0 | 0 | 1 | 30 |
| otu177 | 0 | 0 | 0 | 0 | 0 | 0 | 0 | 0 | 0 | 0 | 0 | 0 | 0 | 0 | 86 | 0 | 0 | 172 |
| otu178 | 21 | 0 | 0 | 0 | 0 | 0 | 0 | 1 | 0 | 0 | 0 | 0 | 0 | 3 | 0 | 0 | 0 | 50 |
| otu179 | 0 | 0 | 0 | 0 | 0 | 0 | 0 | 0 | 0 | 0 | 0 | 0 | 0 | 0 | 34 | 0 | 0 | 68 |
| otu18 | 0 | 4 | 567 | 0 | 0 | 0 | 0 | 0 | 9 | 0 | 0 | 0 | 18 | 45 | 1965 | 0 | 26 | 5268 |
| otu180 | 0 | 0 | 13 | 0 | 0 | 0 | 0 | 0 | 0 | 0 | 0 | 0 | 0 | 0 | 0 | 0 | 0 | 26 |
| otu181 | 1 | 0 | 0 | 0 | 0 | 0 | 0 | 0 | 39 | 0 | 0 | 0 | 0 | 0 | 0 | 2 | 0 | 84 |
| otu182 | 21 | 21 | 3 | 0 | 0 | 0 | 25 | 0 | 0 | 2 | 0 | 0 | 14 | 46 | 0 | 1 | 3 | 272 |
| otu183 | 0 | 0 | 0 | 0 | 0 | 0 | 0 | 0 | 0 | 0 | 0 | 0 | 0 | 0 | 25 | 0 | 0 | 50 |
| otu184 | 0 | 10 | 0 | 0 | 0 | 0 | 0 | 0 | 0 | 0 | 0 | 0 | 0 | 0 | 0 | 0 | 4 | 28 |
| otu185 | 0 | 6 | 0 | 0 | 0 | 0 | 0 | 14 | 0 | 0 | 0 | 0 | 0 | 0 | 0 | 0 | 1 | 42 |
| otu186 | 0 | 0 | 0 | 0 | 0 | 0 | 0 | 0 | 0 | 0 | 0 | 0 | 0 | 0 | 39 | 0 | 0 | 78 |
| otu187 | 0 | 0 | 0 | 0 | 0 | 0 | 0 | 0 | 0 | 0 | 0 | 0 | 0 | 0 | 9 | 0 | 0 | 18 |
| otu188 | 0 | 0 | 0 | 0 | 0 | 0 | 0 | 18 | 0 | 0 | 0 | 0 | 0 | 0 | 0 | 0 | 0 | 36 |
| otu189 | 1 | 0 | 0 | 0 | 0 | 0 | 27 | 0 | 11 | 0 | 0 | 0 | 3 | 1 | 0 | 37 | 0 | 160 |
| otu19 | 0 | 0 | 2427 | 0 | 106 | 0 | 0 | 0 | 12 | 0 | 0 | 0 | 0 | 0 | 31 | 0 | 0 | 5152 |
| otu190 | 0 | 0 | 0 | 0 | 0 | 0 | 0 | 0 | 0 | 0 | 0 | 0 | 0 | 0 | 21 | 0 | 0 | 42 |
| otu191 | 0 | 0 | 0 | 0 | 0 | 0 | 1 | 0 | 0 | 0 | 0 | 0 | 1 | 2 | 0 | 28 | 0 | 64 |
| otu192 | 0 | 0 | 6 | 0 | 0 | 0 | 0 | 0 | 8 | 0 | 0 | 0 | 0 | 0 | 0 | 0 | 0 | 28 |
| otu193 | 1 | 0 | 5 | 0 | 0 | 0 | 0 | 0 | 0 | 0 | 0 | 0 | 5 | 0 | 0 | 17 | 5 | 66 |
| otu194 | 1 | 0 | 5 | 0 | 0 | 0 | 124 | 0 | 10 | 0 | 0 | 0 | 1 | 0 | 0 | 487 | 5 | 1266 |
| otu195 | 0 | 0 | 0 | 0 | 0 | 0 | 0 | 0 | 0 | 0 | 0 | 0 | 0 | 0 | 23 | 0 | 0 | 46 |
| otu196 | 0 | 0 | 0 | 0 | 0 | 0 | 0 | 0 | 0 | 0 | 0 | 0 | 0 | 0 | 0 | 28 | 0 | 56 |
| otu197 | 0 | 0 | 6 | 0 | 0 | 0 | 0 | 0 | 0 | 0 | 0 | 0 | 0 | 0 | 23 | 0 | 0 | 58 |
| otu198 | 0 | 0 | 0 | 0 | 0 | 0 | 0 | 0 | 0 | 0 | 0 | 0 | 0 | 0 | 24 | 0 | 0 | 48 |
| otu199 | 0 | 0 | 0 | 0 | 0 | 0 | 0 | 0 | 0 | 0 | 0 | 0 | 0 | 8 | 0 | 0 | 2 | 20 |
| otu2 | 0 | 0 | 26321 | 0 | 0 | 0 | 0 | 0 | 29 | 64 | 0 | 0 | 0 | 0 | 73 | 0 | 4 | 52982 |
| otu20 | 62 | 10 | 0 | 0 | 0 | 0 | 30 | 378 | 5 | 0 | 0 | 0 | 122 | 406 | 0 | 648 | 65 | 3452 |
| otu200 | 7 | 0 | 0 | 0 | 0 | 0 | 0 | 5 | 2 | 0 | 0 | 0 | 0 | 0 | 0 | 5 | 0 | 38 |
| otu201 | 0 | 0 | 0 | 0 | 0 | 0 | 0 | 0 | 0 | 0 | 0 | 0 | 0 | 0 | 16 | 0 | 0 | 32 |
| otu202 | 0 | 0 | 0 | 0 | 0 | 0 | 0 | 0 | 0 | 0 | 0 | 0 | 0 | 0 | 19 | 0 | 0 | 38 |
| otu203 | 0 | 0 | 0 | 0 | 0 | 0 | 0 | 0 | 0 | 0 | 0 | 0 | 0 | 0 | 30 | 0 | 0 | 60 |
| otu204 | 0 | 0 | 21 | 0 | 0 | 0 | 0 | 0 | 0 | 0 | 0 | 0 | 1 | 189 | 20 | 0 | 63 | 588 |
| otu205 | 0 | 0 | 0 | 0 | 0 | 0 | 0 | 0 | 0 | 0 | 0 | 0 | 0 | 0 | 0 | 48 | 0 | 96 |
| otu206 | 0 | 1 | 0 | 0 | 0 | 0 | 1 | 0 | 7 | 0 | 0 | 0 | 0 | 0 | 1 | 58 | 1 | 138 |
| otu207 | 0 | 0 | 0 | 0 | 0 | 0 | 0 | 0 | 0 | 0 | 0 | 0 | 0 | 0 | 38 | 0 | 0 | 76 |
| otu208 | 0 | 0 | 0 | 0 | 0 | 0 | 18 | 0 | 6 | 0 | 0 | 0 | 0 | 0 | 0 | 17 | 0 | 82 |
| otu209 | 0 | 0 | 0 | 0 | 0 | 0 | 0 | 0 | 0 | 0 | 0 | 0 | 2 | 0 | 0 | 33 | 0 | 70 |
| otu21 | 0 | 0 | 2 | 0 | 0 | 0 | 0 | 0 | 1 | 0 | 0 | 0 | 0 | 0 | 3335 | 0 | 0 | 6676 |
| otu210 | 0 | 0 | 0 | 0 | 0 | 0 | 0 | 0 | 0 | 0 | 0 | 0 | 0 | 0 | 146 | 0 | 0 | 292 |
| otu211 | 0 | 0 | 0 | 0 | 0 | 0 | 1 | 0 | 0 | 0 | 0 | 0 | 16 | 0 | 0 | 35 | 0 | 104 |
| otu212 | 0 | 0 | 0 | 0 | 0 | 0 | 0 | 8 | 0 | 0 | 0 | 0 | 0 | 0 | 0 | 0 | 0 | 16 |
| otu213 | 0 | 0 | 15 | 0 | 0 | 0 | 0 | 0 | 0 | 0 | 0 | 0 | 2 | 3 | 0 | 10 | 1 | 62 |
| otu214 | 3 | 3 | 40 | 0 | 0 | 0 | 16 | 7 | 4 | 0 | 0 | 0 | 30 | 4 | 0 | 423 | 2 | 1064 |
| otu215 | 0 | 0 | 0 | 0 | 0 | 0 | 5 | 0 | 0 | 0 | 0 | 0 | 19 | 0 | 0 | 38 | 0 | 124 |
| otu216 | 0 | 2 | 0 | 0 | 0 | 0 | 0 | 0 | 0 | 0 | 0 | 0 | 0 | 0 | 0 | 34 | 3 | 78 |
| otu217 | 0 | 0 | 0 | 0 | 0 | 0 | 0 | 0 | 0 | 0 | 0 | 0 | 0 | 0 | 27 | 0 | 0 | 54 |
| otu218 | 0 | 0 | 0 | 0 | 0 | 0 | 0 | 0 | 5 | 1 | 0 | 0 | 2 | 2 | 0 | 2 | 0 | 24 |
| otu219 | 0 | 0 | 0 | 0 | 0 | 0 | 0 | 0 | 0 | 0 | 0 | 0 | 0 | 0 | 29 | 0 | 0 | 58 |
| otu22 | 0 | 0 | 2 | 0 | 0 | 0 | 0 | 0 | 1 | 0 | 0 | 0 | 0 | 0 | 1741 | 0 | 0 | 3488 |
| otu220 | 0 | 0 | 0 | 0 | 0 | 0 | 0 | 0 | 0 | 0 | 0 | 0 | 5 | 4 | 0 | 5 | 2 | 32 |
| otu221 | 0 | 0 | 0 | 0 | 0 | 0 | 6 | 0 | 12 | 0 | 0 | 0 | 0 | 0 | 0 | 0 | 0 | 36 |
| otu222 | 1 | 1 | 0 | 0 | 0 | 0 | 2 | 0 | 0 | 0 | 0 | 0 | 0 | 16 | 0 | 26 | 11 | 114 |
| otu223 | 0 | 0 | 60 | 0 | 0 | 0 | 0 | 0 | 0 | 0 | 0 | 0 | 0 | 0 | 0 | 0 | 0 | 120 |
| otu224 | 0 | 0 | 0 | 0 | 0 | 0 | 0 | 0 | 0 | 0 | 0 | 0 | 10 | 0 | 0 | 0 | 0 | 20 |
| otu225 | 0 | 0 | 0 | 0 | 0 | 0 | 0 | 14 | 0 | 0 | 0 | 0 | 0 | 0 | 0 | 0 | 0 | 28 |
| otu226 | 0 | 0 | 0 | 0 | 0 | 0 | 0 | 0 | 0 | 0 | 0 | 0 | 0 | 0 | 1882 | 0 | 0 | 3764 |
| otu227 | 0 | 0 | 1 | 0 | 0 | 0 | 0 | 0 | 5 | 0 | 0 | 0 | 0 | 0 | 8 | 0 | 0 | 28 |
| otu228 | 4 | 3 | 0 | 0 | 0 | 0 | 0 | 0 | 0 | 0 | 0 | 0 | 35 | 3 | 0 | 47 | 13 | 210 |
| otu229 | 0 | 0 | 0 | 0 | 0 | 0 | 0 | 0 | 0 | 0 | 0 | 0 | 0 | 0 | 62 | 0 | 0 | 124 |
| otu23 | 0 | 0 | 1259 | 24 | 0 | 0 | 0 | 0 | 11 | 223 | 0 | 0 | 0 | 0 | 16 | 0 | 1 | 3068 |
| otu230 | 0 | 1 | 0 | 8 | 4 | 6 | 0 | 0 | 0 | 1 | 0 | 3 | 0 | 0 | 0 | 0 | 2 | 50 |
| otu231 | 0 | 0 | 4 | 0 | 0 | 0 | 36 | 0 | 1 | 0 | 0 | 0 | 3 | 0 | 0 | 935 | 0 | 1958 |
| otu232 | 0 | 0 | 0 | 0 | 0 | 0 | 36 | 0 | 0 | 0 | 0 | 0 | 2 | 0 | 0 | 8 | 0 | 92 |
| otu233 | 0 | 0 | 0 | 0 | 0 | 0 | 0 | 0 | 0 | 0 | 0 | 0 | 0 | 0 | 23 | 0 | 0 | 46 |
| otu234 | 0 | 0 | 0 | 0 | 0 | 0 | 0 | 0 | 1 | 0 | 0 | 0 | 2 | 0 | 0 | 49 | 0 | 104 |
| otu235 | 0 | 0 | 9 | 0 | 0 | 0 | 0 | 0 | 0 | 0 | 0 | 0 | 0 | 0 | 0 | 0 | 0 | 18 |
| otu236 | 0 | 0 | 0 | 0 | 0 | 0 | 2 | 0 | 0 | 0 | 0 | 0 | 0 | 0 | 0 | 46 | 0 | 96 |
| otu237 | 0 | 0 | 0 | 0 | 0 | 0 | 0 | 0 | 0 | 0 | 0 | 0 | 0 | 0 | 65 | 0 | 0 | 130 |
| otu238 | 0 | 0 | 0 | 0 | 0 | 0 | 0 | 0 | 0 | 0 | 0 | 0 | 0 | 0 | 7 | 0 | 0 | 14 |
| otu239 | 0 | 0 | 0 | 0 | 0 | 0 | 0 | 16 | 0 | 0 | 0 | 0 | 0 | 0 | 0 | 0 | 0 | 32 |
| otu24 | 0 | 0 | 69 | 0 | 0 | 0 | 0 | 0 | 34 | 0 | 0 | 0 | 0 | 36 | 2445 | 0 | 0 | 5168 |
| otu240 | 0 | 0 | 0 | 0 | 0 | 0 | 17 | 0 | 0 | 0 | 0 | 0 | 0 | 0 | 0 | 0 | 0 | 34 |
| otu241 | 0 | 0 | 0 | 0 | 0 | 0 | 0 | 0 | 0 | 0 | 0 | 0 | 0 | 9 | 0 | 0 | 1 | 20 |
| otu242 | 0 | 0 | 0 | 0 | 0 | 0 | 0 | 0 | 2 | 0 | 0 | 0 | 0 | 0 | 37 | 0 | 0 | 78 |
| otu243 | 0 | 0 | 9 | 0 | 0 | 0 | 0 | 0 | 0 | 0 | 0 | 0 | 0 | 0 | 0 | 0 | 0 | 18 |
| otu244 | 0 | 10 | 29 | 0 | 0 | 0 | 8 | 0 | 1 | 0 | 0 | 0 | 5 | 5 | 1 | 331 | 22 | 824 |
| otu245 | 0 | 0 | 0 | 0 | 0 | 0 | 0 | 0 | 0 | 0 | 0 | 0 | 0 | 0 | 0 | 9 | 0 | 18 |
| otu246 | 0 | 0 | 0 | 0 | 0 | 0 | 7 | 0 | 18 | 0 | 0 | 0 | 0 | 0 | 0 | 30 | 0 | 110 |
| otu247 | 0 | 0 | 0 | 0 | 0 | 0 | 0 | 0 | 0 | 0 | 0 | 0 | 0 | 0 | 21 | 0 | 0 | 42 |
| otu248 | 0 | 0 | 0 | 0 | 0 | 0 | 0 | 0 | 0 | 0 | 0 | 0 | 0 | 0 | 8 | 0 | 0 | 16 |
| otu249 | 0 | 0 | 1 | 0 | 0 | 0 | 0 | 0 | 0 | 0 | 0 | 0 | 0 | 0 | 6 | 0 | 0 | 14 |
| otu25 | 133 | 2 | 0 | 31 | 685 | 14 | 2 | 10 | 6 | 188 | 17 | 27 | 2 | 10 | 0 | 3 | 0 | 2260 |
| otu250 | 0 | 5 | 6 | 0 | 0 | 0 | 5 | 0 | 9 | 0 | 0 | 0 | 0 | 15 | 3 | 28 | 4 | 150 |
| otu251 | 0 | 0 | 0 | 0 | 0 | 0 | 0 | 0 | 7 | 0 | 0 | 0 | 0 | 0 | 6 | 0 | 0 | 26 |
| otu252 | 0 | 0 | 0 | 0 | 0 | 0 | 0 | 0 | 0 | 0 | 0 | 0 | 0 | 0 | 7 | 0 | 0 | 14 |
| otu253 | 0 | 0 | 4 | 0 | 0 | 0 | 0 | 0 | 0 | 0 | 0 | 0 | 0 | 0 | 9 | 0 | 0 | 26 |
| otu254 | 0 | 0 | 6 | 0 | 0 | 0 | 7 | 0 | 2 | 0 | 0 | 0 | 6 | 20 | 0 | 132 | 0 | 346 |
| otu255 | 0 | 0 | 0 | 0 | 0 | 0 | 0 | 0 | 0 | 0 | 0 | 0 | 0 | 0 | 89 | 0 | 0 | 178 |
| otu256 | 0 | 5 | 59 | 0 | 0 | 0 | 12 | 0 | 1 | 0 | 0 | 0 | 7 | 10 | 0 | 624 | 16 | 1468 |
| otu258 | 0 | 0 | 1 | 0 | 0 | 0 | 0 | 0 | 0 | 0 | 0 | 0 | 0 | 0 | 10 | 0 | 0 | 22 |
| otu259 | 1 | 2 | 13 | 0 | 0 | 0 | 28 | 0 | 1 | 0 | 0 | 0 | 7 | 0 | 0 | 684 | 0 | 1472 |
| otu26 | 0 | 0 | 4 | 0 | 0 | 0 | 0 | 0 | 4 | 0 | 0 | 0 | 3 | 345 | 1465 | 0 | 101 | 3844 |
| otu260 | 4 | 1 | 0 | 0 | 0 | 0 | 1 | 7 | 2 | 4 | 0 | 0 | 4 | 7 | 0 | 8 | 0 | 76 |
| otu261 | 0 | 0 | 0 | 0 | 0 | 0 | 0 | 0 | 0 | 0 | 0 | 0 | 0 | 0 | 52 | 0 | 0 | 104 |
| otu262 | 0 | 0 | 0 | 1 | 1 | 0 | 1 | 1 | 0 | 0 | 0 | 6 | 1 | 0 | 0 | 2 | 2 | 30 |
| otu263 | 0 | 0 | 0 | 0 | 0 | 0 | 0 | 0 | 21 | 0 | 0 | 0 | 0 | 0 | 533 | 0 | 0 | 1108 |
| otu264 | 0 | 0 | 1 | 0 | 0 | 0 | 2 | 0 | 1 | 0 | 0 | 0 | 0 | 2 | 0 | 30 | 0 | 72 |
| otu265 | 0 | 0 | 0 | 0 | 0 | 0 | 0 | 0 | 0 | 0 | 0 | 0 | 6 | 0 | 0 | 5 | 3 | 28 |
| otu267 | 0 | 5 | 0 | 0 | 0 | 0 | 0 | 0 | 0 | 0 | 0 | 0 | 0 | 0 | 0 | 0 | 0 | 10 |
| otu268 | 0 | 0 | 0 | 0 | 0 | 0 | 0 | 0 | 13 | 0 | 0 | 0 | 0 | 0 | 0 | 0 | 0 | 26 |
| otu269 | 0 | 0 | 0 | 0 | 0 | 0 | 0 | 0 | 0 | 0 | 0 | 0 | 0 | 13 | 0 | 59 | 10 | 164 |
| otu27 | 0 | 0 | 700 | 0 | 0 | 0 | 0 | 0 | 5 | 63 | 0 | 0 | 0 | 0 | 692 | 0 | 0 | 2920 |
| otu28 | 0 | 0 | 9 | 0 | 0 | 0 | 0 | 0 | 11 | 0 | 0 | 0 | 0 | 0 | 1591 | 0 | 0 | 3222 |
| otu29 | 239 | 1 | 4 | 0 | 0 | 0 | 120 | 4 | 2 | 0 | 0 | 0 | 73 | 0 | 1 | 615 | 0 | 2118 |
| otu3 | 362 | 5267 | 673 | 0 | 0 | 0 | 2265 | 3 | 1869 | 77 | 3 | 3 | 256 | 3096 | 282 | 14222 | 6878 | 70512 |
| otu30 | 109 | 199 | 1 | 0 | 11 | 1 | 124 | 38 | 3 | 34 | 0 | 0 | 4 | 15 | 2 | 273 | 85 | 1798 |
| otu31 | 0 | 0 | 4 | 0 | 0 | 0 | 0 | 0 | 0 | 0 | 0 | 0 | 0 | 0 | 572 | 0 | 0 | 1152 |
| otu32 | 0 | 0 | 1 | 0 | 0 | 0 | 0 | 0 | 6 | 0 | 0 | 0 | 0 | 0 | 468 | 0 | 0 | 950 |
| otu33 | 0 | 0 | 0 | 0 | 0 | 0 | 0 | 0 | 0 | 0 | 0 | 0 | 0 | 0 | 1197 | 0 | 0 | 2394 |
| otu34 | 0 | 0 | 0 | 0 | 0 | 0 | 0 | 0 | 0 | 0 | 0 | 0 | 0 | 0 | 1123 | 0 | 0 | 2246 |
| otu35 | 0 | 0 | 0 | 0 | 0 | 0 | 0 | 0 | 0 | 0 | 0 | 0 | 0 | 0 | 1195 | 0 | 0 | 2390 |
| otu36 | 0 | 0 | 0 | 0 | 0 | 0 | 0 | 0 | 0 | 0 | 0 | 0 | 0 | 0 | 696 | 0 | 0 | 1392 |
| otu37 | 2 | 47 | 30 | 0 | 0 | 0 | 10 | 0 | 1 | 0 | 0 | 1 | 10 | 287 | 2 | 758 | 351 | 2998 |
| otu38 | 6 | 153 | 25 | 0 | 0 | 0 | 184 | 0 | 68 | 0 | 0 | 0 | 13 | 88 | 7 | 966 | 64 | 3148 |
| otu39 | 598 | 4 | 0 | 0 | 0 | 0 | 0 | 0 | 0 | 0 | 0 | 0 | 3 | 18 | 0 | 7 | 3 | 1266 |
| otu4 | 0 | 1 | 95 | 0 | 0 | 0 | 0 | 0 | 323 | 0 | 0 | 0 | 7 | 0 | 30560 | 0 | 6 | 61984 |
| otu40 | 1 | 0 | 3 | 0 | 0 | 0 | 653 | 0 | 33 | 0 | 0 | 0 | 3 | 1 | 3 | 426 | 0 | 2246 |
| otu41 | 46 | 1 | 13 | 0 | 0 | 0 | 1 | 0 | 582 | 0 | 0 | 0 | 8 | 0 | 0 | 6 | 1 | 1316 |
| otu42 | 0 | 0 | 0 | 0 | 0 | 0 | 0 | 0 | 4 | 0 | 0 | 0 | 5 | 289 | 0 | 14 | 83 | 790 |
| otu43 | 0 | 0 | 0 | 0 | 0 | 0 | 0 | 0 | 2 | 0 | 0 | 0 | 0 | 0 | 310 | 0 | 0 | 624 |
| otu44 | 0 | 0 | 5 | 0 | 0 | 0 | 0 | 0 | 45 | 0 | 0 | 0 | 0 | 0 | 336 | 0 | 0 | 772 |
| otu45 | 8 | 3 | 7 | 40 | 376 | 0 | 1 | 3 | 4 | 12 | 0 | 9 | 3 | 10 | 125 | 2 | 4 | 1214 |
| otu46 | 0 | 0 | 2 | 0 | 0 | 0 | 0 | 0 | 8 | 0 | 0 | 0 | 0 | 0 | 256 | 0 | 0 | 532 |
| otu47 | 0 | 0 | 1 | 1 | 0 | 0 | 38 | 0 | 9 | 0 | 0 | 0 | 0 | 0 | 278 | 0 | 0 | 654 |
| otu48 | 0 | 0 | 0 | 0 | 0 | 0 | 0 | 0 | 0 | 0 | 0 | 0 | 0 | 0 | 878 | 0 | 0 | 1756 |
| otu49 | 1 | 16 | 27 | 0 | 0 | 0 | 421 | 0 | 218 | 0 | 0 | 0 | 8 | 15 | 27 | 908 | 7 | 3296 |
| otu5 | 112 | 288 | 1144 | 0 | 0 | 6 | 167 | 161 | 85 | 0 | 0 | 0 | 1691 | 186 | 13 | 5663 | 380 | 19792 |
| otu50 | 0 | 11 | 15 | 0 | 0 | 0 | 0 | 63 | 0 | 0 | 0 | 0 | 0 | 0 | 2 | 232 | 21 | 688 |
| otu51 | 0 | 1 | 0 | 0 | 0 | 0 | 7 | 13 | 4 | 0 | 0 | 0 | 4 | 0 | 0 | 507 | 5 | 1082 |
| otu52 | 6 | 0 | 0 | 10 | 105 | 1 | 1 | 2 | 15 | 0 | 12 | 8 | 1 | 9 | 0 | 0 | 1 | 342 |
| otu53 | 30 | 0 | 4 | 0 | 0 | 0 | 8 | 0 | 3 | 0 | 2 | 0 | 2 | 223 | 0 | 266 | 62 | 1200 |
| otu54 | 0 | 0 | 0 | 0 | 0 | 0 | 0 | 0 | 0 | 0 | 0 | 0 | 0 | 0 | 616 | 0 | 0 | 1232 |
| otu55 | 0 | 0 | 2 | 0 | 0 | 0 | 0 | 0 | 18 | 0 | 0 | 0 | 1 | 8 | 519 | 0 | 0 | 1096 |
| otu56 | 0 | 0 | 0 | 0 | 0 | 0 | 0 | 0 | 0 | 0 | 0 | 0 | 0 | 0 | 809 | 0 | 0 | 1618 |
| otu57 | 0 | 0 | 0 | 0 | 0 | 0 | 0 | 0 | 9 | 0 | 0 | 0 | 0 | 0 | 193 | 0 | 0 | 404 |
| otu58 | 0 | 0 | 0 | 0 | 0 | 0 | 0 | 316 | 0 | 0 | 0 | 0 | 0 | 0 | 0 | 0 | 1 | 634 |
| otu59 | 0 | 0 | 0 | 0 | 0 | 0 | 0 | 0 | 0 | 0 | 0 | 0 | 0 | 0 | 550 | 0 | 0 | 1100 |
| otu6 | 274 | 20 | 112 | 8 | 0 | 0 | 6949 | 63 | 473 | 0 | 0 | 21 | 310 | 53 | 73 | 11948 | 28 | 40664 |
| otu60 | 0 | 2 | 0 | 0 | 0 | 0 | 7 | 2 | 2 | 0 | 0 | 0 | 0 | 0 | 0 | 202 | 3 | 436 |
| otu61 | 0 | 0 | 2 | 0 | 0 | 0 | 0 | 0 | 0 | 0 | 0 | 0 | 0 | 0 | 153 | 0 | 0 | 310 |
| otu62 | 0 | 221 | 0 | 0 | 0 | 0 | 0 | 0 | 0 | 0 | 0 | 0 | 1 | 0 | 0 | 0 | 48 | 540 |
| otu63 | 0 | 0 | 11 | 0 | 0 | 0 | 0 | 0 | 3 | 0 | 0 | 0 | 0 | 7 | 312 | 0 | 0 | 666 |
| otu64 | 0 | 0 | 20 | 0 | 0 | 0 | 0 | 0 | 1 | 0 | 0 | 0 | 0 | 0 | 348 | 0 | 0 | 738 |
| otu65 | 0 | 0 | 176 | 0 | 0 | 0 | 0 | 0 | 1 | 0 | 0 | 0 | 0 | 0 | 0 | 0 | 0 | 354 |
| otu66 | 0 | 0 | 23 | 0 | 0 | 0 | 0 | 0 | 9 | 0 | 0 | 0 | 0 | 0 | 56 | 6 | 1 | 190 |
| otu67 | 0 | 0 | 6 | 0 | 0 | 0 | 0 | 0 | 10 | 0 | 0 | 0 | 0 | 0 | 463 | 0 | 0 | 958 |
| otu68 | 0 | 0 | 38 | 0 | 0 | 0 | 0 | 0 | 0 | 0 | 0 | 0 | 0 | 0 | 240 | 0 | 0 | 556 |
| otu69 | 0 | 0 | 28 | 0 | 0 | 0 | 0 | 0 | 73 | 0 | 0 | 0 | 0 | 0 | 48 | 0 | 7 | 312 |
| otu7 | 0 | 423 | 0 | 1 | 2598 | 1188 | 0 | 0 | 0 | 0 | 0 | 0 | 0 | 0 | 0 | 0 | 316 | 9052 |
| otu70 | 0 | 0 | 3 | 0 | 0 | 0 | 0 | 0 | 22 | 0 | 0 | 4 | 0 | 0 | 108 | 0 | 0 | 274 |
| otu71 | 8 | 2 | 0 | 0 | 0 | 0 | 0 | 2 | 0 | 0 | 0 | 0 | 2 | 0 | 0 | 189 | 7 | 420 |
| otu72 | 0 | 57 | 0 | 0 | 0 | 0 | 0 | 0 | 0 | 0 | 0 | 0 | 0 | 0 | 0 | 0 | 46 | 206 |
| otu73 | 3 | 0 | 2 | 0 | 0 | 0 | 21 | 0 | 3 | 0 | 0 | 0 | 11 | 0 | 0 | 376 | 0 | 832 |
| otu74 | 0 | 0 | 0 | 0 | 0 | 0 | 0 | 0 | 0 | 0 | 0 | 0 | 0 | 0 | 277 | 0 | 0 | 554 |
| otu75 | 1 | 0 | 9 | 0 | 0 | 0 | 219 | 0 | 6 | 0 | 0 | 0 | 4 | 0 | 1 | 147 | 0 | 774 |
| otu76 | 0 | 0 | 0 | 0 | 0 | 0 | 0 | 0 | 0 | 74 | 0 | 0 | 0 | 0 | 0 | 0 | 0 | 148 |
| otu77 | 0 | 0 | 0 | 0 | 0 | 0 | 0 | 0 | 1 | 0 | 0 | 0 | 0 | 0 | 68 | 0 | 0 | 138 |
| otu78 | 0 | 0 | 0 | 0 | 0 | 0 | 5 | 0 | 0 | 0 | 0 | 0 | 46 | 0 | 165 | 3 | 0 | 438 |
| otu79 | 0 | 0 | 0 | 0 | 0 | 0 | 0 | 0 | 1 | 0 | 0 | 0 | 0 | 0 | 173 | 0 | 0 | 348 |
| otu8 | 20 | 25 | 1 | 0 | 0 | 0 | 78 | 182 | 25 | 0 | 0 | 0 | 2913 | 976 | 1 | 332 | 245 | 9596 |
| otu80 | 0 | 0 | 0 | 0 | 0 | 0 | 0 | 0 | 1 | 0 | 0 | 0 | 0 | 0 | 77 | 0 | 0 | 156 |
| otu81 | 0 | 0 | 0 | 5 | 0 | 0 | 1 | 0 | 0 | 0 | 0 | 0 | 3 | 0 | 188 | 6 | 0 | 406 |
| otu82 | 1 | 65 | 0 | 0 | 0 | 0 | 14 | 0 | 3 | 0 | 0 | 0 | 0 | 3 | 0 | 74 | 29 | 378 |
| otu83 | 72 | 3 | 0 | 0 | 0 | 0 | 0 | 0 | 0 | 0 | 0 | 0 | 0 | 1 | 0 | 0 | 1 | 154 |
| otu84 | 0 | 0 | 0 | 0 | 0 | 0 | 0 | 0 | 0 | 0 | 0 | 0 | 0 | 0 | 165 | 0 | 0 | 330 |
| otu85 | 0 | 0 | 55 | 0 | 0 | 0 | 0 | 0 | 4 | 0 | 0 | 0 | 0 | 0 | 0 | 0 | 1 | 120 |
| otu86 | 0 | 0 | 1 | 0 | 0 | 0 | 31 | 0 | 77 | 2 | 0 | 0 | 6 | 5 | 2 | 20 | 0 | 288 |
| otu87 | 0 | 0 | 0 | 0 | 0 | 0 | 0 | 0 | 8 | 0 | 0 | 0 | 0 | 0 | 48 | 0 | 0 | 112 |
| otu88 | 0 | 1 | 1 | 0 | 0 | 0 | 0 | 18 | 8 | 0 | 0 | 0 | 0 | 26 | 0 | 10 | 8 | 144 |
| otu89 | 26 | 13 | 1 | 0 | 0 | 0 | 2 | 13 | 1 | 0 | 0 | 0 | 36 | 41 | 0 | 4 | 19 | 312 |
| otu9 | 59 | 32 | 3846 | 0 | 0 | 0 | 107 | 271 | 12 | 53 | 0 | 0 | 390 | 71 | 1 | 3509 | 113 | 16928 |
| otu90 | 0 | 0 | 0 | 0 | 0 | 0 | 0 | 0 | 0 | 0 | 0 | 0 | 0 | 0 | 83 | 0 | 0 | 166 |
| otu91 | 0 | 0 | 0 | 0 | 0 | 0 | 0 | 0 | 0 | 0 | 0 | 0 | 0 | 0 | 197 | 0 | 0 | 394 |
| otu92 | 3 | 110 | 1 | 0 | 0 | 0 | 3 | 0 | 0 | 0 | 0 | 0 | 5 | 17 | 0 | 52 | 135 | 652 |
| otu93 | 0 | 0 | 0 | 0 | 0 | 0 | 0 | 0 | 1 | 0 | 0 | 0 | 0 | 0 | 113 | 0 | 0 | 228 |
| otu94 | 0 | 0 | 2 | 0 | 0 | 0 | 0 | 0 | 1 | 0 | 0 | 0 | 0 | 74 | 0 | 0 | 63 | 280 |
| otu95 | 0 | 0 | 1 | 0 | 0 | 0 | 0 | 0 | 2 | 0 | 0 | 0 | 1 | 0 | 97 | 0 | 0 | 202 |
| otu96 | 0 | 0 | 0 | 0 | 0 | 0 | 0 | 0 | 0 | 0 | 0 | 0 | 0 | 0 | 253 | 0 | 0 | 506 |
| otu97 | 0 | 0 | 0 | 0 | 0 | 0 | 0 | 0 | 0 | 0 | 0 | 0 | 0 | 0 | 141 | 0 | 0 | 282 |
| otu98 | 0 | 0 | 0 | 0 | 0 | 0 | 1 | 0 | 3 | 0 | 0 | 0 | 0 | 0 | 62 | 3 | 0 | 138 |
| otu99 | 0 | 0 | 0 | 0 | 0 | 0 | 0 | 0 | 0 | 0 | 0 | 0 | 0 | 0 | 305 | 0 | 0 | 610 |
