# Supplementary material for: Endophytic and epiphytic metabarcoding reveals fungal communities on cashew phyllosphere in Kenya
Source: PLoS One. 2024 Jul 17;19(7):e0305600. doi: 10.1371/journal.pone.0305600 (PMC11253924; doi:10.1371/journal.pone.0305600)
Supplement: S3 File — (DOCX) [file pone.0305600.s003.docx]

**S3 Table**

| SampleID | klbf.1 | klbl.1 | klbr.1 | klfl.1 | klfr.1 | kll.1 | kwbf.1 | kwbl.1 | kwbr.1 | kwfl.1 | kwfr.1 | kwl.1 | lmbf.1 | lmbl.1 | lmbr.1 | lmfl.1 | lml.1 | Total_OTUs |
| --- | --- | --- | --- | --- | --- | --- | --- | --- | --- | --- | --- | --- | --- | --- | --- | --- | --- | --- |
| otu1 | 0 | 0 | 280 | 0 | 0 | 0 | 0 | 0 | 349 | 0 | 0 | 0 | 2 | 6986 | 126877 | 0 | 2241 | 136735 |
| otu10 | 222 | 140 | 196 | 0 | 0 | 4 | 45 | 288 | 18 | 98 | 0 | 7 | 394 | 5754 | 7 | 3111 | 1322 | 11606 |
| otu100 | 0 | 0 | 0 | 0 | 0 | 0 | 1 | 0 | 3 | 0 | 0 | 0 | 0 | 0 | 46 | 0 | 0 | 50 |
| otu101 | 0 | 0 | 112 | 0 | 0 | 0 | 0 | 0 | 0 | 0 | 0 | 0 | 0 | 0 | 0 | 0 | 0 | 112 |
| otu102 | 0 | 0 | 0 | 0 | 0 | 0 | 0 | 0 | 0 | 0 | 0 | 0 | 0 | 0 | 146 | 0 | 0 | 146 |
| otu103 | 0 | 0 | 2 | 0 | 0 | 0 | 0 | 0 | 1 | 0 | 0 | 0 | 0 | 74 | 0 | 0 | 63 | 140 |
| otu104 | 0 | 0 | 1 | 0 | 0 | 0 | 0 | 0 | 2 | 0 | 0 | 0 | 1 | 0 | 95 | 0 | 0 | 99 |
| otu106 | 34 | 0 | 0 | 0 | 0 | 0 | 1 | 0 | 0 | 0 | 0 | 4 | 1 | 0 | 0 | 7 | 0 | 47 |
| otu107 | 0 | 0 | 0 | 0 | 0 | 0 | 0 | 0 | 0 | 0 | 0 | 0 | 0 | 0 | 213 | 0 | 0 | 213 |
| otu108 | 0 | 0 | 13 | 0 | 0 | 0 | 0 | 0 | 2 | 0 | 0 | 0 | 0 | 44 | 150 | 0 | 69 | 278 |
| otu109 | 0 | 0 | 0 | 0 | 0 | 0 | 0 | 0 | 10 | 0 | 0 | 0 | 0 | 0 | 109 | 0 | 0 | 119 |
| otu11 | 779 | 75 | 16 | 14 | 50 | 0 | 873 | 104 | 358 | 113 | 1 | 8 | 181 | 374 | 26 | 1391 | 16 | 4379 |
| otu110 | 0 | 0 | 1 | 0 | 0 | 0 | 95 | 0 | 10 | 0 | 0 | 0 | 4 | 0 | 0 | 49 | 0 | 159 |
| otu111 | 0 | 0 | 0 | 0 | 0 | 0 | 0 | 0 | 3 | 0 | 0 | 0 | 0 | 0 | 52 | 0 | 0 | 55 |
| otu112 | 0 | 0 | 13 | 0 | 0 | 0 | 0 | 0 | 0 | 0 | 0 | 0 | 0 | 17 | 130 | 0 | 0 | 160 |
| otu113 | 0 | 0 | 78 | 0 | 0 | 0 | 24 | 0 | 9 | 0 | 0 | 0 | 20 | 1 | 0 | 249 | 0 | 381 |
| otu114 | 0 | 5 | 58 | 0 | 0 | 0 | 13 | 0 | 1 | 0 | 0 | 0 | 6 | 10 | 0 | 624 | 15 | 732 |
| otu115 | 0 | 0 | 0 | 0 | 0 | 0 | 0 | 0 | 0 | 0 | 0 | 0 | 0 | 0 | 132 | 0 | 0 | 132 |
| otu116 | 0 | 0 | 0 | 0 | 0 | 0 | 0 | 0 | 0 | 0 | 0 | 0 | 0 | 0 | 96 | 0 | 0 | 96 |
| otu117 | 9 | 9 | 0 | 0 | 0 | 0 | 0 | 68 | 7 | 0 | 0 | 0 | 0 | 12 | 1 | 14 | 2 | 122 |
| otu118 | 0 | 0 | 0 | 0 | 0 | 0 | 0 | 0 | 0 | 0 | 0 | 0 | 0 | 0 | 87 | 0 | 0 | 87 |
| otu12 | 174 | 20 | 101 | 0 | 0 | 3 | 264 | 17 | 72 | 0 | 0 | 0 | 536 | 108 | 9 | 7489 | 27 | 8820 |
| otu120 | 0 | 0 | 0 | 0 | 0 | 0 | 0 | 0 | 0 | 0 | 0 | 0 | 0 | 0 | 100 | 0 | 0 | 100 |
| otu121 | 0 | 0 | 4 | 0 | 0 | 0 | 0 | 0 | 0 | 0 | 0 | 0 | 0 | 0 | 83 | 0 | 0 | 87 |
| otu122 | 0 | 0 | 47 | 0 | 0 | 0 | 0 | 0 | 0 | 0 | 0 | 0 | 0 | 0 | 1 | 0 | 0 | 48 |
| otu123 | 0 | 0 | 0 | 0 | 0 | 0 | 0 | 0 | 0 | 0 | 0 | 0 | 0 | 0 | 0 | 28 | 0 | 28 |
| otu124 | 0 | 0 | 0 | 0 | 0 | 0 | 0 | 0 | 0 | 0 | 0 | 0 | 0 | 0 | 234 | 0 | 0 | 234 |
| otu126 | 0 | 0 | 3 | 0 | 0 | 0 | 11 | 0 | 1 | 0 | 0 | 0 | 6 | 0 | 0 | 93 | 0 | 114 |
| otu127 | 0 | 0 | 0 | 4 | 33 | 0 | 0 | 0 | 0 | 0 | 0 | 0 | 0 | 0 | 0 | 0 | 0 | 37 |
| otu128 | 0 | 0 | 81 | 0 | 0 | 0 | 0 | 0 | 0 | 0 | 0 | 0 | 0 | 0 | 0 | 0 | 0 | 81 |
| otu13 | 131 | 3 | 1092 | 0 | 0 | 0 | 6 | 2 | 27 | 59 | 0 | 0 | 12 | 612 | 232 | 172 | 54 | 2402 |
| otu14 | 0 | 0 | 9 | 0 | 0 | 0 | 0 | 0 | 15 | 0 | 0 | 0 | 0 | 0 | 7270 | 0 | 0 | 7294 |
| otu15 | 0 | 0 | 0 | 0 | 0 | 0 | 0 | 0 | 1 | 0 | 0 | 0 | 0 | 0 | 3428 | 0 | 0 | 3429 |
| otu16 | 14 | 12 | 88 | 15 | 0 | 0 | 0 | 0 | 124 | 0 | 0 | 0 | 134 | 0 | 1437 | 351 | 9 | 2184 |
| otu17 | 0 | 0 | 344 | 0 | 0 | 0 | 1 | 0 | 72 | 0 | 0 | 0 | 0 | 0 | 1238 | 12 | 0 | 1667 |
| otu19 | 0 | 0 | 2425 | 0 | 106 | 0 | 0 | 0 | 12 | 0 | 0 | 0 | 0 | 0 | 31 | 0 | 0 | 2574 |
| otu20 | 62 | 10 | 0 | 0 | 0 | 0 | 30 | 378 | 5 | 0 | 0 | 0 | 120 | 406 | 0 | 639 | 65 | 1715 |
| otu21 | 0 | 0 | 2 | 0 | 0 | 0 | 0 | 0 | 1 | 0 | 0 | 0 | 0 | 0 | 3285 | 0 | 0 | 3288 |
| otu22 | 0 | 0 | 1259 | 24 | 0 | 0 | 0 | 0 | 11 | 223 | 0 | 0 | 0 | 0 | 16 | 0 | 1 | 1534 |
| otu23 | 0 | 0 | 2 | 0 | 0 | 0 | 0 | 0 | 1 | 0 | 0 | 0 | 0 | 0 | 1741 | 0 | 0 | 1744 |
| otu24 | 0 | 0 | 69 | 0 | 0 | 0 | 0 | 0 | 34 | 0 | 0 | 0 | 0 | 36 | 2401 | 0 | 0 | 2540 |
| otu25 | 133 | 2 | 0 | 31 | 685 | 14 | 2 | 10 | 6 | 188 | 17 | 27 | 2 | 10 | 0 | 3 | 0 | 1130 |
| otu27 | 0 | 0 | 700 | 0 | 0 | 0 | 0 | 0 | 5 | 63 | 0 | 0 | 0 | 0 | 692 | 0 | 0 | 1460 |
| otu28 | 239 | 1 | 4 | 0 | 0 | 0 | 120 | 4 | 2 | 0 | 0 | 0 | 73 | 0 | 1 | 615 | 0 | 1059 |
| otu3 | 362 | 5267 | 673 | 0 | 0 | 0 | 2265 | 3 | 1869 | 77 | 3 | 3 | 256 | 3096 | 282 | 14222 | 6878 | 35256 |
| otu32 | 8 | 3 | 7 | 40 | 376 | 0 | 1 | 3 | 4 | 12 | 0 | 9 | 3 | 10 | 125 | 2 | 4 | 607 |
| otu33 | 0 | 0 | 4 | 0 | 0 | 0 | 0 | 0 | 0 | 0 | 0 | 0 | 0 | 0 | 573 | 0 | 0 | 577 |
| otu34 | 0 | 0 | 1 | 0 | 0 | 0 | 0 | 0 | 6 | 0 | 0 | 0 | 0 | 0 | 468 | 0 | 0 | 475 |
| otu35 | 0 | 0 | 0 | 0 | 0 | 0 | 0 | 0 | 0 | 0 | 0 | 0 | 0 | 0 | 1120 | 0 | 0 | 1120 |
| otu37 | 0 | 0 | 0 | 0 | 0 | 0 | 0 | 0 | 0 | 0 | 0 | 0 | 0 | 0 | 1191 | 0 | 0 | 1191 |
| otu38 | 2 | 47 | 30 | 0 | 0 | 0 | 10 | 0 | 1 | 0 | 0 | 1 | 10 | 287 | 2 | 767 | 350 | 1507 |
| otu39 | 0 | 0 | 0 | 0 | 0 | 0 | 0 | 0 | 0 | 0 | 0 | 0 | 0 | 0 | 660 | 0 | 0 | 660 |
| otu41 | 1 | 0 | 3 | 0 | 0 | 0 | 653 | 0 | 33 | 0 | 0 | 0 | 3 | 1 | 3 | 426 | 0 | 1123 |
| otu42 | 0 | 0 | 0 | 0 | 0 | 0 | 0 | 0 | 4 | 0 | 0 | 0 | 5 | 289 | 0 | 14 | 83 | 395 |
| otu43 | 46 | 1 | 13 | 0 | 0 | 0 | 1 | 0 | 582 | 0 | 0 | 0 | 8 | 0 | 0 | 6 | 1 | 658 |
| otu44 | 0 | 0 | 4 | 0 | 0 | 0 | 0 | 0 | 43 | 0 | 0 | 0 | 0 | 0 | 233 | 0 | 0 | 280 |
| otu45 | 0 | 0 | 0 | 0 | 0 | 0 | 0 | 0 | 2 | 0 | 0 | 0 | 0 | 0 | 310 | 0 | 0 | 312 |
| otu47 | 0 | 0 | 2 | 0 | 0 | 0 | 0 | 0 | 8 | 0 | 0 | 0 | 0 | 0 | 256 | 0 | 0 | 266 |
| otu48 | 0 | 0 | 0 | 0 | 0 | 0 | 0 | 316 | 0 | 0 | 0 | 0 | 0 | 0 | 0 | 0 | 1 | 317 |
| otu49 | 0 | 0 | 0 | 0 | 0 | 0 | 0 | 0 | 0 | 0 | 0 | 0 | 0 | 0 | 848 | 0 | 0 | 848 |
| otu5 | 112 | 288 | 1141 | 0 | 0 | 6 | 162 | 161 | 85 | 0 | 0 | 0 | 1674 | 186 | 13 | 5392 | 379 | 9599 |
| otu50 | 0 | 11 | 14 | 0 | 0 | 0 | 0 | 62 | 0 | 0 | 0 | 0 | 0 | 0 | 2 | 227 | 20 | 336 |
| otu51 | 1 | 16 | 27 | 0 | 0 | 0 | 421 | 0 | 218 | 0 | 0 | 0 | 8 | 15 | 27 | 904 | 7 | 1644 |
| otu52 | 0 | 1 | 0 | 0 | 0 | 0 | 7 | 13 | 4 | 0 | 0 | 0 | 4 | 0 | 0 | 510 | 5 | 544 |
| otu53 | 30 | 0 | 4 | 0 | 0 | 0 | 8 | 0 | 3 | 0 | 2 | 0 | 2 | 223 | 0 | 266 | 62 | 600 |
| otu54 | 0 | 0 | 0 | 0 | 0 | 0 | 0 | 0 | 0 | 0 | 0 | 0 | 0 | 0 | 733 | 0 | 0 | 733 |
| otu55 | 6 | 0 | 0 | 10 | 105 | 1 | 1 | 2 | 15 | 0 | 12 | 8 | 1 | 9 | 0 | 0 | 1 | 171 |
| otu58 | 0 | 2 | 0 | 0 | 0 | 0 | 7 | 2 | 2 | 0 | 0 | 0 | 0 | 0 | 0 | 202 | 3 | 218 |
| otu59 | 0 | 0 | 0 | 0 | 0 | 0 | 0 | 0 | 0 | 0 | 0 | 0 | 0 | 0 | 573 | 0 | 0 | 573 |
| otu6 | 273 | 20 | 114 | 8 | 0 | 0 | 6950 | 63 | 473 | 0 | 0 | 21 | 309 | 53 | 73 | 12013 | 28 | 20398 |
| otu60 | 0 | 0 | 0 | 0 | 0 | 0 | 0 | 0 | 9 | 0 | 0 | 0 | 0 | 0 | 193 | 0 | 0 | 202 |
| otu61 | 0 | 0 | 11 | 0 | 0 | 0 | 0 | 0 | 3 | 0 | 0 | 0 | 0 | 7 | 312 | 0 | 0 | 333 |
| otu62 | 0 | 0 | 0 | 0 | 0 | 0 | 0 | 0 | 0 | 0 | 0 | 0 | 0 | 0 | 564 | 0 | 0 | 564 |
| otu63 | 0 | 0 | 2 | 0 | 0 | 0 | 0 | 0 | 0 | 0 | 0 | 0 | 0 | 0 | 153 | 0 | 0 | 155 |
| otu64 | 0 | 0 | 20 | 0 | 0 | 0 | 0 | 0 | 1 | 0 | 0 | 0 | 0 | 0 | 348 | 0 | 0 | 369 |
| otu65 | 0 | 0 | 172 | 0 | 0 | 0 | 0 | 0 | 1 | 0 | 0 | 0 | 0 | 0 | 0 | 0 | 0 | 173 |
| otu66 | 0 | 221 | 0 | 0 | 0 | 0 | 0 | 0 | 0 | 0 | 0 | 0 | 1 | 0 | 0 | 0 | 48 | 270 |
| otu67 | 0 | 0 | 6 | 0 | 0 | 0 | 0 | 0 | 10 | 0 | 0 | 0 | 0 | 0 | 463 | 0 | 0 | 479 |
| otu69 | 0 | 0 | 23 | 0 | 0 | 0 | 0 | 0 | 9 | 0 | 0 | 0 | 0 | 0 | 56 | 6 | 1 | 95 |
| otu70 | 1 | 0 | 9 | 0 | 0 | 0 | 219 | 0 | 6 | 0 | 0 | 0 | 4 | 0 | 1 | 147 | 0 | 387 |
| otu71 | 3 | 1 | 0 | 0 | 0 | 0 | 0 | 2 | 0 | 0 | 0 | 0 | 0 | 0 | 0 | 173 | 0 | 179 |
| otu72 | 0 | 0 | 28 | 0 | 0 | 0 | 0 | 0 | 73 | 0 | 0 | 0 | 0 | 0 | 48 | 0 | 7 | 156 |
| otu74 | 0 | 0 | 3 | 0 | 0 | 0 | 0 | 0 | 22 | 0 | 0 | 4 | 0 | 0 | 108 | 0 | 0 | 137 |
| otu75 | 0 | 57 | 0 | 0 | 0 | 0 | 0 | 0 | 0 | 0 | 0 | 0 | 0 | 0 | 0 | 0 | 46 | 103 |
| otu76 | 0 | 0 | 0 | 0 | 0 | 0 | 0 | 0 | 0 | 0 | 0 | 0 | 0 | 0 | 259 | 0 | 0 | 259 |
| otu77 | 0 | 0 | 0 | 0 | 0 | 0 | 0 | 0 | 0 | 74 | 0 | 0 | 0 | 0 | 0 | 0 | 0 | 74 |
| otu78 | 11 | 0 | 0 | 0 | 0 | 0 | 109 | 0 | 39 | 0 | 1 | 0 | 1 | 4 | 3 | 17 | 0 | 185 |
| otu8 | 59 | 32 | 3847 | 0 | 0 | 0 | 106 | 271 | 12 | 53 | 0 | 0 | 389 | 71 | 1 | 3491 | 113 | 8445 |
| otu81 | 0 | 0 | 0 | 0 | 0 | 0 | 0 | 0 | 1 | 0 | 0 | 0 | 0 | 0 | 173 | 0 | 0 | 174 |
| otu82 | 0 | 0 | 0 | 0 | 0 | 0 | 0 | 0 | 1 | 0 | 0 | 0 | 0 | 0 | 68 | 0 | 0 | 69 |
| otu83 | 0 | 0 | 0 | 0 | 0 | 0 | 5 | 0 | 0 | 0 | 0 | 0 | 46 | 0 | 165 | 3 | 0 | 219 |
| otu84 | 0 | 0 | 0 | 0 | 0 | 0 | 0 | 0 | 1 | 0 | 0 | 0 | 0 | 0 | 77 | 0 | 0 | 78 |
| otu85 | 1 | 65 | 0 | 0 | 0 | 0 | 14 | 0 | 3 | 0 | 0 | 0 | 0 | 3 | 0 | 74 | 29 | 189 |
| otu86 | 0 | 0 | 0 | 5 | 0 | 0 | 1 | 0 | 0 | 0 | 0 | 0 | 3 | 0 | 188 | 6 | 0 | 203 |
| otu87 | 0 | 0 | 1 | 0 | 0 | 0 | 31 | 0 | 77 | 2 | 0 | 0 | 6 | 5 | 2 | 20 | 0 | 144 |
| otu88 | 0 | 0 | 2 | 0 | 0 | 0 | 0 | 0 | 0 | 0 | 0 | 0 | 0 | 0 | 189 | 0 | 0 | 191 |
| otu89 | 72 | 3 | 0 | 0 | 0 | 0 | 0 | 0 | 0 | 0 | 0 | 0 | 0 | 1 | 0 | 0 | 1 | 77 |
| otu9 | 20 | 25 | 1 | 0 | 0 | 0 | 78 | 182 | 25 | 0 | 0 | 0 | 2913 | 976 | 1 | 332 | 245 | 4798 |
| otu90 | 0 | 0 | 0 | 0 | 0 | 0 | 0 | 0 | 0 | 0 | 0 | 0 | 0 | 0 | 149 | 0 | 0 | 149 |
| otu91 | 0 | 0 | 55 | 0 | 0 | 0 | 0 | 0 | 4 | 0 | 0 | 0 | 0 | 0 | 0 | 0 | 1 | 60 |
| otu92 | 0 | 0 | 0 | 0 | 0 | 0 | 0 | 0 | 0 | 0 | 0 | 0 | 0 | 0 | 141 | 0 | 0 | 141 |
| otu93 | 0 | 1 | 1 | 0 | 0 | 0 | 0 | 18 | 8 | 0 | 0 | 0 | 0 | 26 | 0 | 10 | 8 | 72 |
| otu94 | 26 | 13 | 1 | 0 | 0 | 0 | 2 | 13 | 1 | 0 | 0 | 0 | 36 | 40 | 0 | 4 | 19 | 155 |
| otu95 | 0 | 0 | 0 | 0 | 0 | 0 | 0 | 0 | 0 | 0 | 0 | 0 | 0 | 0 | 310 | 0 | 0 | 310 |
| otu96 | 0 | 0 | 0 | 0 | 0 | 0 | 0 | 0 | 8 | 0 | 0 | 0 | 0 | 0 | 48 | 0 | 0 | 56 |
| otu97 | 3 | 110 | 1 | 0 | 0 | 0 | 3 | 0 | 0 | 0 | 0 | 0 | 5 | 17 | 0 | 52 | 135 | 326 |
| otu98 | 0 | 0 | 0 | 0 | 0 | 0 | 0 | 0 | 0 | 0 | 0 | 0 | 0 | 0 | 83 | 0 | 0 | 83 |
| otu99 | 0 | 0 | 0 | 0 | 0 | 0 | 0 | 0 | 1 | 0 | 0 | 0 | 0 | 0 | 113 | 0 | 0 | 114 |
