# Supplementary material for: Endophytic and epiphytic metabarcoding reveals fungal communities on cashew phyllosphere in Kenya
Source: PLoS One. 2024 Jul 17;19(7):e0305600. doi: 10.1371/journal.pone.0305600 (PMC11253924; doi:10.1371/journal.pone.0305600)
Supplement: Attachment — Submitted filename: Responses to reviewers.docx [file pone.0305600.s004.docx]

**Responses to reviewers' comments**

| **Reviewer** | **Comment** | **Response** |
| --- | --- | --- |
| **Reviewer #1** |  |  |
|  | Please provide details in the M&M section, such as the replications of each tissue sample; they should be sufficiently supplied. | Tissue samples (Leaf, flower and fruit) were collected across the three study sites. DNA was acquired from each sample in duplicate. However, concerning our study objectives that was anchored on creating insights into the abundance and composition of available, we pooled the DNA together creating a represantive of 18 samples. In the manuscript, the sample collection per county has been indicated and the grand total stated. |
|  | What is the portion of your DNA regarding microbes and plants? How to deal with this in this study? | The microbes DNA (fungal gDNA) was of interest. Concerning the study goals, the use of universal fungal barcodes was a priority, therefore we used primers targeting the ITS and 28S rRNA for fungal DNA therefore excluding the plant DNA. |
|  | Could the term “phyllosphere” cover your study about different plant organs? | Phyllosphere generally refers to all above surface plant organs and therefore in using this word I have specified the key areas of interest in this work, these are (Leaf, flower and fruit). |
|  | The authors use both ITS and 28S rRNA to barcode the presence of the fungi in and on the plants. Why you use this approach? What is the pros and cons? How to interpret the difference? It is better to further discussed in this article | The ITS and 28S rRNA microbe detection vary in rational to abundance and composition because they are in different databases which are updated differently. In addition 28S rRNA database is very recent therefore it is less enriched than ITS however it has notable microbes that are not present in ITS as revealed in this work. This approach is ideal in maximising on detection of available microbial communities that is insightful by creating a plethora of information about microbial communities present. However, the cost incurred tend to limit in addition to sometimes having similar composition although the abundance will always differ. This is important to have the entire databases enriched and updated so the future studies. |
|  | Other suggestions | The other suggestions made have been acted upon and the coherence of content flow established |
| **Reviewer #2** | Address Abstract, Introduction, Discussion and Conclusion sections | In response to the feedback provided, the key sections have been addressed as directed and the changes are trackable in the main manuscript having track changes. Additionally, the need for clarity and precision of the work therein the manuscript has been adhered. |
